# A rapid improved multiplex ligation detection reaction method for the identification of gene mutations in hereditary hearing loss

**DOI:** 10.1371/journal.pone.0215212

**Published:** 2019-04-11

**Authors:** Yalan Liu, Chang Hu, Chang Liu, Deyuan Liu, Lingyun Mei, Chufeng He, Lu Jiang, Hong Wu, Hongsheng Chen, Yong Feng

**Affiliations:** 1 Department of Otolaryngology, Xiangya Hospital, Central South University, Changsha, Hunan, China; 2 Province Key Laboratory of Otolaryngology Critical Diseases, Changsha, Hunan, China; 3 Department of Otolaryngology, The First Hospital of Changsha, Changsha, Hunan, China; 4 R&D Department, Genesky Diagnostics Inc., Suzhou, Jiangsu, China; King Abdulaziz University Hospital, SAUDI ARABIA

## Abstract

Hearing loss (HL) is a common sensory disorder. More than half of HL cases can be attributed to genetic causes. There is no effective therapy for genetic HL at present, early diagnosis to reduce the incidence of genetic HL is important for clinical intervention in genetic HL. Previous studies have identified 111 nonsyndromic hearing loss genes. The most frequently mutated genes identified in NSHL patients in China include GJB2, SLC26A4, and the mitochondrial gene MT-RNR1. It is important to develop HL gene panels in Chinese population, which allow for etiologic diagnosis of both SHL and NSHL. In this study, a total of 220 unrelated Han Chinese patients with bilateral progressive SNHL and 50 unrelated healthy controls were performed Single nucleotide polymorphism (SNP) genotyping using an improved multiplex ligation detection reaction (iMLDR) technique, is to simultaneously detect a total of 32 mutations in ten HL genes, covering all currently characterized mutations involved in the etiology of nonsyndromic or syndromic hearing loss in the Chinese population. The 49 positive samples with known mutations were successfully detected using the iMLDR Technique. For 171 SNHL patients, gene variants were found in 57 cases (33.33%), among which, 30 patients carried mutations in GJB2, 14 patients carried mutations in SLC26A4, seven patients carried mutations in GJB3, and six patients carried mutations in MT-RNR1. The molecular etiology of deafness was confirmed in 12.9% (22/171) of patients carried homozygous variants. These results were verified by Sanger sequencing, indicating that the sensitivity and specificity of the iMLDR technique was 100%. We believe that the implementation of this population-specific technology at an efficient clinical level would have great value in HL diagnosis and treatment.

## Introduction

Hearing loss (HL) is a common sensory disorder, affecting 360 million people worldwide (WHO, 2017) and more than 27 million individuals in China alone. It is estimated that the prevalence of HL is 1 to 3 per 1000 live births, and the total number of children that suffer from HL exceeds 32 million worldwide (WHO, 2017). Both genetic and environmental factors can lead to HL. More than half of congenital HL cases can be attributed to genetic causes, of which 30% are syndromic (SHL) and 70% are nonsyndromic (NSHL). HL is commonly caused by variation in a single gene that adheres to a simple Mendelian inheritance pattern: autosomal dominant, autosomal recessive, X-linked inheritance, Y-linked inheritance, or maternal inheritance. However, more than 1000 known variants in over 100 genes cause HL. Previous studies have mapped 165 NSHL loci, and 111 of these have been cloned (http://hereditaryhearingloss.org, March 2018).

There is no effective therapy for genetic HL at present. Therefore, early diagnosis to reduce the incidence of genetic HL is important for clinical intervention in genetic HL. Currently, many cases of HL are identified at birth via newborn hearing screening (NBHS), which generally includes either otoacoustic emission (OAE) or auditory brainstem response (ABR) testing or both. However, many HL cases only become apparent later in life with late-onset hearing impairment mutations or following an environmental insult, such as noise exposure, antibiotic use, or head trauma. In these cases, the implications of missed and delayed diagnoses indicate an opportunity to utilize the newest ideas and tools of genetic screening for improved diagnostic measures.

The previous standard of practice indicated the need for genetic screening in HL only in cases of suspected congenital sensorineural HL (SNHL) or for individuals that fail the NBHS [1]. Application of this standard resulted in only about one-third of patients with HL receiving a definitive molecular diagnosis after having undergone comprehensive genetic testing for known HL genes [2]. In addition, in the field of HL, unique genetic profiles are being identified in specific ethnic groups at an increasing rate. In different countries or ethnic groups, and in different regions of the same country, the frequency of mutations varies greatly [3–5]. Epidemiological survey data indicate that the most frequently mutated genes identified in patients with NSHL in China include *GJB2* (OMIM: 121011), *SLC26A4* (OMIM: 605646), and the mitochondrial gene *MT-RNR1* (OMIM: 561000) and that each of these genes have multiple common mutations. These data provide a theoretical basis for the large-scale development of deafness gene screening and diagnosis in various genomic regions [6, 7]. Therefore, rare genes or mutations that are not commonly included in panels designed for diagnosis of genetic HL in multiple populations, or ethnic groups, can be included in customized, population-specific, gene panels. Additionally, it is important to develop HL gene panels that allow for etiologic diagnosis of both SHL and NSHL.

Several screening methods, each with their own advantages, can be used to screen for the causative mutations of HL. These approaches include Sanger sequencing, PCR-restriction fragment length polymorphism (PCR-RFLP), real-time PCR, SNaPshot, matrix-assisted laser desorption/ionization–time-of-flight mass spectrometry (MALDI-TOF MS), and microarray analysis. However, these approaches are time-consuming, tedious, costly, and not suitable for large-scale detection in clinical applications. In this study, we introduce a newly developed multiplex genetic screening system, called the iMLDR technique, to investigate neonatal HL samples in the Chinese population. The objective of this screening system is to simultaneously detect a total of 32 mutations in the *GJB2*, *SLC26A4*, and *MT-RNR1* genes, covering all currently characterized mutations involved in the etiology of nonsyndromic or syndromic SNHL in the Chinese population.

## Materials and methods

### Samples

A total of 220 unrelated Han Chinese patients with bilateral progressive SNHL were recruited between 2011 and 2013 from the Otolaryngology Department of Xiangya Hospital, Central South University. We recruited patients in whom deafness resulted from unclear causative factors. Patients in who clear causative factors for deafness were identified such as noise exposure, trauma (exception for the patients who are identified with enlarged vestibular aqueduct by medical imaging), intrauterine infection, poisoning, or tumors were excluded. A detailed medical history was available for each proband. Every participant was examined thoroughly, including systemic and specialized physical examination, electric otoscopy, and audiological assessment animation. All participants were probands and were divided into 215 cases of nonsyndromic SNHL and 5 cases of WS based on clinical history, physical examination, audiological examination, and imaging tests. Among these samples, 49 cases (33 prelingual and 16 postlingual) were already tested by Sanger sequencing and were used to validate the performance of the iMLDR technique (**Table 1**). Of these, 32 patients were from family and 17 were from sporadic cases. The controls consisted of 50 unrelated healthy Chinese volunteers with normal hearing and without another genetic disease.

**Table 1:** Forty-nine positive cases used to validate the performance of the iMLDR technique.

Gene	Chromosome	Nucleotide Change	Heterozygous	Homozygous
***GJB2***	**13**	c.139G>T	2	0
		c.176_191del16	1	0
		c.235delC	10	6
		c.299_300delAT	2	0
		c.571T>G	1	0
***SLC26A4***	**7**	IVS7-2A>G	3	3
		c.1174A>T	1	0
		c.1229C>T	2	0
		c.2168A>G	3	0
***MT-RNR1***	**mtDNA**	c.1555A>G	-	11
***GJB3***	**1**	c.580G>A	1	0
***PAX3***	**2**	c.812G>A	1	0
***MITF***	**3**	c.650G>T	1	0
***CEACAM16***	**19**	c.505G>A	1	0

Genomic DNA was extracted from 10 ml peripheral blood using standard phenol-chloroform protocols and stored at -20°C[8]. Written informed consent was obtained from all patients and controls, and the study was approved by the Human Ethics Committee of Central South University and is compliant with the Code of Ethics of the World Medical Association[9].

### Selection of mutations responsible for hereditary HL (HHL)

We searched the literature available on the human gene mutation database[10], PubMed, Embase, the Chinese National Knowledge Infrastructure, and the Chinese Wanfang Literature Database to estimate the frequency of mutations in causative genes for HL in the Chinese population. We then selected an initial set of genes, which had been previously implicated in HL, for inclusion in our study. The mutation list included single-nucleotide changes and deletions. The 32 mutations chosen included all currently characterized mutations involved in the etiology of nonsyndromic and syndromic SNHL (Table 2). Mutations in the mutation list included those involving mitochondrial and autosomal deafness genes, 28 NSHL variants and 4 common SHL variants related to Waardenburg syndrome (WS), two maternally inherited variants, and one X-linked variant.

**Table 2:** Thirty-two mutations responsible for hereditary hearing loss.

Gene	Chromosome	Nucleotide change	Allelic Change	dbSNP rs#
***GJB2***	**13**	IVS1+1G>A	G/A	rs80338940
		c.35 del G	G/-	rs80338939
		c.71G>A	G/A	rs104894396
		c.139G>T	G/T	rs104894398
		c.167delT	T/-	rs80338942
		c.176_191del16	gctgcaagaacgtgtg/-	
		c.235delC	C/-	rs80338943
		c.299_300delAT	AT/-	
		c.571T>G	T/C	
		c.596C>T	C/T	
***GJB3***	**1**	c.538C>T	C/T	rs74315319
		c.547 G>A	G/A	
		c.580G>A	G/A	rs121908852
***SLC26A4***	**7**	c.281C>T	C/T	
		IVS7-2A>G	A/G	
		c.1174A>T	A/T	
		c.1226G>A	G/A	
		c.1229C>T	C/T	
		IVS15+5G>A	G/A	
		c.2027T>A	T/A	
		c.2168A>G	A/G	rs121908362
***MT-RNR1***	**mtDNA**	c.1494C>T	C/T	
		c.1555A>G	A/G	
***COCH***	**14**	c.151C>T	C/T	rs28938175
		c.1625G>A,c.1625G>T	G/A;G/T	rs121908933
***POU3F4***	**X**	c.967C>G	C/G	rs104894924
***PAX3***	**2**	c.812G>A	G/A	
***MITF***	**3**	c.650G>T	G/T	
		c.648_650delAAG	AAG/-	
***SOX10***	**22**	c.113delG	G/-	
***CEACAM16***	**19**	c.505G>A	G/A	
		c.418A>C	A/C	

rs#: reference SNP cluster

### Single nucleotide polymorphism genotyping using the iMLDR technique

**Fig 1:**
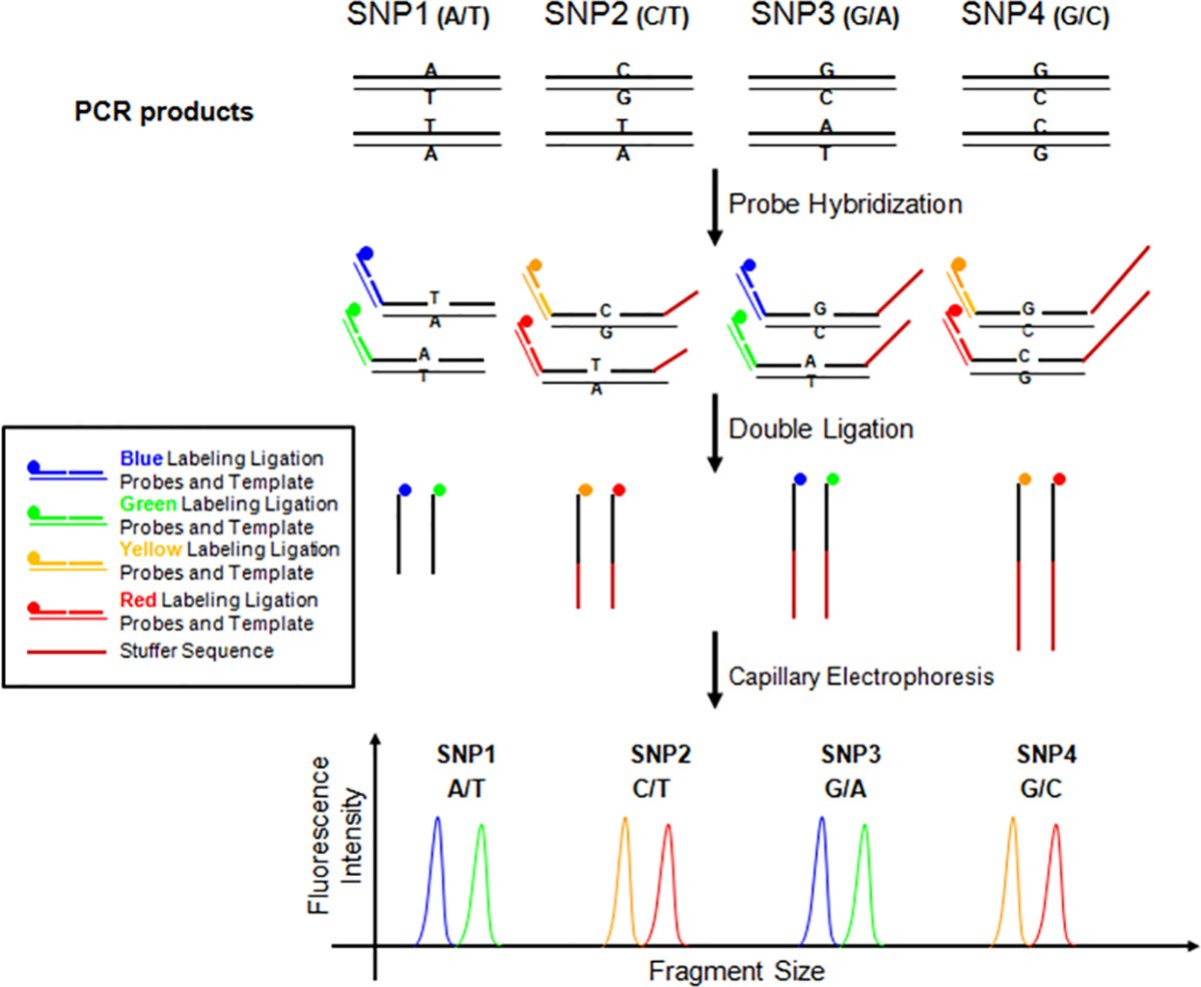
Illustration for Multiplex SNP Genotyping Using iMLDR technique.

Single nucleotide polymorphism (SNP) genotyping was performed using an improved multiplex ligation detection reaction (iMLDR) technique recently developed by Genesky Biotechnologies Inc. (Shanghai, China). The basic principle of this technique is illustrated in Fig 1. Generally, for each SNP locus, three 5´-phosphorylated probes were designed, two allele-specific 5’ probes, and one common 3’ probe. Each 5’ probe was designed with a dye-specific sequence at the 5’ end and an allele-specific sequence at 3’ end. A variable length stuffer sequence may be added at the 3’ end of the 3’ probes to distinguish ligation products with identical dye labeling. The 3’ end of the allele-specific 5’ probe can hybridize to the target genomic DNA and ligate to the adjoining 3’ probe. The 5’ half can hybridize to an oligo template and ligate to a corresponding 5’ dye-labeled oligo, making the allele-specific 5’ probe dye-labeled. This system means that an effective ligation product will be produced by a double ligation consisting of a labeling ligation and an allelic discrimination ligation. The two allele-specific 5’ probes contain different 5’ dye-specific sequence to enable labeling with different dyes by the labeling ligation. The sequence of the two allele-specific 5’ probes differs at the 3’ end and will ligate to the 3’ probe when bound to the corresponding allele DNA template. Therefore, the ligation products from two alleles will be labeled with different dyes. In Fig 1, four dyes (blue, green, yellow, and red) are used to distinguish the four ligation products from two SNP loci with same size. This approach can be significantly scaled-up to increase the number of SNP loci assayed in one reaction.

We applied the iMLDR technique to genotype 32 SNP loci in one ligation reaction. A multiplex PCR reaction was designed to amplify 18 fragments covering all 32 SNP loci. The 20 μl PCR reaction mixture contained 1 x GC Buffer I (Takara), 3.0 mM Mg^2+^, 0.3 mM dNTPs, 1 U of Hot-Start Taq DNA polymerase (Takara), 1 μl of primer mixture, and 20 ng of genomic DNA. Primer sequences and concentrations are described in **S1 Table**. The PCR cycling conditions were as follows: 95°C for 2 min; followed by 11 cycles of 94°C for 20 s, 65–0.5°C/cycle for 40 s, and 72°C 1 min 30 s; then 24 cycles of 94°C for 20 s, 59°C for 30 s, and 72°C 1 min 30 s; and a final extension of 72°C for 2 min and holding at 4°C. The PCR products were equally mixed and purified by digestion with 1 U of shrimp alkaline phosphatase at 37°C for 1 hr, followed by deactivation of the phosphatase at 75°C for 15 min. The 20 μl ligation reaction contained 1 x ligation buffer, 80 U Taq DNA ligase (NEB), 1 μl of labeling oligo mixture, 2 μl of probe mixture, and 5 μl of purified PCR product mixture. The oligo and probe information is described in **S2** and **S3 Tables**, respectively. The cycling program for the ligation reaction was 95°C 2 min followed by 38 cycles of 94°C for 1 min and 56°C for 4 min and a hold at 4°C. Ligation product (0.5 μl) was loaded into an ABI 3730xl DNA analyzer and the raw data were analyzed by GeneMapper 4.1. All primers, probes, and labeling oligos were designed by and ordered from Genesky Biotechnologies Inc. (Shanghai, China).

### Sanger sequencing

Detected variants were confirmed by Sanger sequencing and data were analyzed using the DNASTAR software program (DNASTAR, Inc., Madison, Wisconsin, US).

## Results

### Performance of iMLDR Technique

The 49 positive patients utilized in this study were initially characterized by Sanger Sequencing. Samples with known mutations were successfully detected using the iMLDR Technique. These samples included five compound heterozygous variants detected in nine patients (**Table 3)**,which were inherited from the parents. The iMLDR findings consistent with the initial results obtained using an array. Three *GJB2* heterozygous (c.176_191del16, c.235delC and c.299_300delAT) and two *SLC26A4* heterozygous (IVS7-2A>G) mutations were detected in 50 controls (**Table 4**). The assay demonstrated 100% sensitivity and specificity.

**Table 3:** Compound Heterozygous detected in 49 positive cases.

Compound Heterozygous	No. of Samples
SLC26A4_ IVS7-2A>G/ c.2168A>G	1
GJB2_ c.235delC/ c.299_300delAT	2
GJB2_ c.235delC/ MT-RNR1_ c.1555A>G	1
GJB2_ c.235delC/ SLC26A4_ IVS7-2A>G	1
GJB3_ c.580G>A/ MT-RNR1_ c.1555A>G	1
GJB2_ c.571T>G/SLC26A4_ c.1174A>T	1
SLC26A4_ c.1229C>T/ c.2168A>G	2

**Table 4:** Variants detected in 50 controls.

Gene	Nucleotide change	Heterozygous	Frequency
			n = 50
***GJB2***	c.176_191del16	1	2%
	c.235delC	1	2%
	c.299_300delAT	1	2%
***SLC26A4***	IVS7-2A>G	2	4%

### Mutations detected by iMLDR in a cohort of bilateral progressive SNHL patients

For 171 bilateral progressive SNHL patients, gene variants were found in 57 cases (33.33%) **(Table 5)**. In summary, 30 patients carried mutations in *GJB2*, 14 patients carried mutations in *SLC26A4*, seven patients carried mutations in *GJB3*, and six patients carried mutations in *MT-RNR1*. Of these variants, nine were compound heterozygous **(Table 6)**, 35 were heterozygous (20.47%), and 22 were homozygous variants (12.87%). Mutations in *GJB2*, *SLC26A4*, and *GJB3* exhibit autosomal recessive inheritance. Therefore, only SNHL patients with homozygous or compound heterozygous pathological mutations would present with hearing impairment caused by the mutations in these genes. Since the 31 patients whose parents’ DNA was not available, We cannot be sure that the nine compound heterozygous mutations of the patients are located in one allele or two alleles respectively, which needs further analysis. Through this analysis, the molecular etiology of deafness was confirmed in 12.9% (22/171) of patients carried homozygous variants. To clarify the genetic etiology for the 35 patients who carry heterozygous variants, Next-Generation Sequencing(NGS) based techniques to analyze the entire coding sequence of the involved genes could be performed to find out the possible existence of a second mutant allele.

**Table 5:** Variants detected in 171 SNHL patients.

Gene	Chromosome	Nucleotide	Homozygous	Heterozygous	Frequency
		change			n = 171
***GJB2***	**13**	c.176_191del16	0	1	0.58%
		c.235delC	12	4	9.36%
		c.299_300delAT	2	9	64.33%
		c.571T>G	0	2	1.17%
***GJB3***	**1**	c.538C>T	0	1	0.58%
		c.580G>A	0	6	3.50%
***SLC26A4***	**7**	IVS7-2A>G	2	4	3.50%
		c.1229C>T	0	4	2.34%
		IVS15+5G>A	0	1	0.58%
		c.2168A>G	0	3	1.75%
***MT-RNR1***	**mtDNA**	1494 C>T	1	0	0.58%
		c.1555A>G	5	0	2.92%

**Table 6:** Compound Heterozygous detected in 171 SNHL patients.

Compound Heterozygous	No. of Samples
SLC26A4_ IVS7-2A>G/ c.1229C>T	1
SLC26A4_ IVS7-2A>G/ c.2168A>G	1
GJB2_ c.235delC/ c.176_191del16	1
GJB2_ c.235delC/ c.299_300delAT	6

*GJB2* was the most prevalent causative gene among patients with nonsyndromic SNHL and the frequency of *GJB2* mutations was 17.54% (30/171). Three mutations, c.235delC, c.299_300delAT, and c.176_191del16, were detected in *GJB2*. The most prevalent mutation was c.235delC, which accounted for 53.33% (16/30) of all mutant *GJB2* alleles. SLC26A4 was the second most prevalent causative gene among the nonsyndromic SNHL patients examined (8.19%, 14/171). Four distinct mutations, IVS7-2A>G, c.1229C>T, IVS15+5G>A, and c.2168A>G, were detected in *SLC26A4*. IVS7-2A>G was the most prevalent mutation, accounting for 42.86% (6/14) of all mutant *SLC26A4* alleles. These results were verified by Sanger sequencing, indicating that the accuracy rate of the iMLDR technique was 100%.

## Discussion

HL affects one in 1000 newborns[11]. However, the heterogeneity of etiologies leading to HL provides a challenge for effective diagnosis and treatment. Mutations in *GJB2*, *SLC26A4*, and the mitochondrial gene *MT-RNR1* were the most frequently identified in NSHL patients. *GJB2* accounts for about 50% of NSHL cases. To date, up to 350 *GJB2* mutations have been reported (http://www.hgmd.cf.ac.uk). The most frequently reported mutation in *GJB2* in East Asian populations is c.235delC[12, 13]. In this study, *GJB2* was the most prevalent causative gene among nonsyndromic SNHL patients. In this gene, three mutations, c.235delC, c.299_300delAT, and c.176_191del16, were detected. Consistent with the results of previous c.235delC was the most prevalent *GJB2* mutation detected in this study [14]. The autosomal recessive inheritance of *GJB2* mutations means that only the NSHL patients with homozygous or compound heterozygous pathological *GJB2* mutations would present with hearing impairment. Therefore, monoallelic *GJB2* mutations could not explain the molecular etiology of the subject's HL in this study. Additional mutations, in other alleles, might be responsible for the HL phenotype. Alternatively, other factors, such as congenital cytomegalovirus (CMV) infection or additional environmental factors, could contribute to HL in this study population[15].

The second most common cause of HHL identified in this study was mutations in the *SLC26A4* gene, which accounted for 5–10% of prelingual HL. The types and frequencies of mutations in *SLC26A4* are differ based on ethnicity. In Chinese HL patients, the carrier frequency of the *SCL26A4* c.919-2A>G mutation accounts for 69.1% of all cases associated with *SLC26A4* mutation [16, 17]. In European populations, *SLC26A4* c.1246A>C and IVS8+1G>A are the two most common mutations [18]. *SLC26A4* was the second most prevalent causative gene among the nonsyndromic SNHL patients examined in this study. Four distinct *SLC26A4* mutations, IVS7-2A>G, c.1229C>T, IVS15+5G>A, and c.2168A>G, were detected, with IVS7-2A>G the most prevalent. *SLC26A4* is the causative gene for enlarged vestibular aqueduct (EVA), and more than 300 *SLC26A4* variants have been identified in cases with EVA (www.healthcare.uiowa.edu/labs/pendredandbor). Routine clinical examinations to diagnose EVA involve audiological tests and temporal bone imaging to reveal the expansile vestibular or endolymphatic sac. EVA manifests clinically as fluctuating or progressive SNHL, ranging from mild to profound deafness[19], and most patients are diagnosed when their hearing is already poor. Therefore, early clinical genetic diagnoses of EVA patients are critical to implement the appropriate disease control and prevention responses, such as avoiding head trauma, getting cold, and noise stimulation. Moreover, when patients with HL are diagnosed with EVA, they can choose hearing aids or artificial cochlear implantation in a timely manner. Currently studies suggest that a *SLC26A4* biallelic variant (compound heterozygous or homozygous) is the main cause of EVA. EVA patients carrying *SLC26A4* biallelic variants usually can be verified by videography diagnosis[20]. In this study, 12 cases with monoallelic *SLC26A4* variants were identified and may only be carriers. In these cases, additional mutations in other alleles may be responsible for the HL phenotype. There could be other undetected mutations in *SLC26A4* regions not examined in this study such as the promoter region, or in a potential intron splice site. Alternatively, there might be a digenic pattern of inheritance implicating a second gene in the etiology of HL in these patients [21]. Furthermore, the interaction between genetic and environmental factors may play a role in the pathogenic process of EVA[22].

Mitochondrial 1555A>G and 1494 C>T mutations that lead to toxic deafness are a common cause of genetic HL in the Chinese population, accounting for 5% of prelingual HL[23, 24]. In this study, 2.92% (5/171) of patients with nonsyndromic SNHL were homoplastic for 1555A>G. Moreover, another patient was found to harbor the homozygous mutation in 12S rRNA 1494 C>T. This suggests that mitochondrial 1555A>G and 1494 C>T mutations might be common in Chinese HL populations and that screening for these two mutations should be performed routinely in molecular-diagnostic centers. It was previously shown that the penetrance of the A1555G mutation can be enhanced by treatment with aminoglycosides. The identification of the A1555G mutation in families with deafness, the presymptomatic detection of this mutation in maternally related subjects, and the avoidance of aminoglycosides by individuals who are positive for the A1555G mutation should help in the prevention of deafness.

In HHL, roughly 70% of cases are nonsyndromic and 30% of prelingual deafness cases are associated with > 400 types of syndromes [25]. The most common autosomal-dominant syndromic deafness is WS, which is characterized by sensorineural deafness, distinctive facial features, and pigment disturbances. WS is clinically and genetically highly heterogeneous. Four subtypes of WS, WS1 (OMIM: 193500), WS2 (OMIM: 193510), WS3 (OMIM: 148820), and WS4 (OMIM: 277580) have been classified based on the presence or absence of additional symptoms. Heterozygous *PAX3* (OMIM: 606597) mutations were responsible for 90% of WS1 patients, and WS3 is caused by heterozygous or homozygous *PAX3* mutations [26]. Fifteen percent of patients with WS2 carried heterozygous *MITF* (OMIM: 156845) mutations [27]. *SOX10* (OMIM: 602229) deletions were identified in WS2 patients, and we identified two novel *SOX10* mutations in the first report of WS4 in Chinese patients [28, 29]. In this study, we included one *PAX3* mutation (c.812G>A), two *MITF* mutations (c.650G>T and c.648_650delAAG), and one *SOX10* mutation (c.113delG) detected previously in Chinese patients with WS in the panel. The inclusion of these mutations enabled our diagnostic technology to simultaneously cover nonsyndromic deafness and syndromic deafness.

We also included another nine mutations in our screening panel. In 1998, Xia. et al. cloned the *GJB3* gene, which encodes human gap junction protein β, which was responsible for bilateral high-frequency hearing impairment [30]. *COCH* (OMIM: 603196) is the most frequently reported gene responsible for autosomal dominant nonsyndromic progressive SNHL [31–33]. *POU3F4* (OMIM: 300039) is the most common gene responsible for X-linked HHL [34]. Using exome sequencing, we identified a novel *CEACAM16* mutation associated with autosomal dominant NSHL, DFNA4B, in a Chinese family. This is the first report in China, and the second report in the literature, of a family with autosomal dominant NSHL caused by a *CEACAM16* mutation. The mutations included in this panel represent the genes that are most frequently involved in deafness, most of which have been reported more than twice in the Chinese population. There was no mutations detected in *COCH*, *POU3F4*, *PAX3*, *MITF*, *SOX10* and *CEACAM16* in this study. The reason may be that the incidence of these six genes is not very high in HHL patients. Larger sample screening studies should be performed in the future.

In our study, we sought to establish a new molecular method based on the iMLDR technique for identification of HHL mutations that, on the one hand, would be capable of simultaneously genotyping of 32 SNP loci with high accuracy and reliability and, on the other hand, would be simple enough for application even in routine laboratories. We used Sanger sequencing to confirm our results, and found that the false positive and the false negative rates were both 0%. We confirmed that in 22 of 171 deaf patients, HL was due to nonsyndromic hereditary deafness, and that 22 cases might be attributed to genetic factors. To clarify the genetic etiology for the 35 patients who carry heterozygous variants and the patients who do not bear any of these mutations, Next-Generation Sequencing(NGS) based gene panels to analyze the entire coding sequence of all the known HHL involved genes, or Whole-exome sequencing (WES) by targeting the protein-coding regions of the genome, could be performed to find out the possible existence of mutant alleles. Additionally, since molecular confirmation of the causative variations for SNHL is important for genetic counseling of patients, steps should be taken to ensure patient safety and confidentiality through several available guidelines, such as through the guideline of American College of Medical Genetics and Genomics (ACMG). Compared with other screening technologies such as NGS, the iMLDR technology has advantage of low cost that the screening costs is less than 0.3 cents/SNP locus, which can be suggested as the initial screening technique of SNHL mutation screening. And then if necessary, the NGS based techniques covering all known genes for SNHL could be further performed. In the field of HL, unique genetic variations were identified in specific ethnic groups. As a result, rare genes or mutations that are not commonly included in panels meant for multi-population use can be included in customized population-specific gene panels. Compared with disease-specific but large-scale NGS gene panels to unearth common HL gene variants, this new technology developed in this study can be stratified by ethnic group or geography and applied clinically in smaller, more targeted HHL diagnostic panels, as the multiplex PCR reaction can be designed to amplify different fragments covering different SNP loci. We believe that the implementation of this population-specific technology at an efficient clinical level would have great value in HL diagnosis and treatment.

## Supporting information

S1 Table Primer sequences and concentration in PCR mixture 1. (DOCX)

S2 Table Oligo sequences and concentration in labeling oligo mixture. (DOCX)

S3 Table Probe sequences and concentration in probe mixture. (DOCX)

## References

[1] Liming BJ, Carter J, Cheng A, Choo D, Curotta J, Carvalho D, et al International Pediatric Otolaryngology Group (IPOG) consensus recommendations: Hearing loss in the pediatric patient. Int J Pediatr Otorhinolaryngol. 2016;90:251–8. Epub 2016/10/13. S0165-5876(16)30316-0 [pii] 10.1016/j.ijporl.2016.09.016 . 27729144

[2] Sommen M, Wuyts W, Van Camp G. Molecular diagnostics for hereditary hearing loss in children. Expert Rev Mol Diagn. 2017;17(8):751–60. Epub 2017/06/09. 10.1080/14737159.2017.1340834 . 28593790

[3] Tekin M, Arici ZS. Genetic epidemiological studies of congenital/prelingual deafness in Turkey: population structure and mating type are major determinants of mutation identification. Am J Med Genet A. 2007;143A(14):1583–91. Epub 2007/04/21. 10.1002/ajmg.a.31702 . 17444514

[4] Angeli S, Lin X, Liu XZ. Genetics of hearing and deafness. Anat Rec (Hoboken). 2012;295(11):1812–29. Epub 2012/10/10. 10.1002/ar.22579 23044516 PMC4523052

[5] Qing J, Yan D, Zhou Y, Liu Q, Wu W, Xiao Z, et al Whole-exome sequencing to decipher the genetic heterogeneity of hearing loss in a Chinese family with deaf by deaf mating. PLoS One. 2014;9(10):e109178 Epub 2014/10/08. 10.1371/journal.pone.0109178 PONE-D-14-13958 [pii]. 25289672 PMC4188603

[6] Dai P, Yu F, Han B, Liu X, Wang G, Li Q, et al GJB2 mutation spectrum in 2,063 Chinese patients with nonsyndromic hearing impairment. J Transl Med. 2009;7:26 Epub 2009/04/16. 1479-5876-7-26 [pii] 10.1186/1479-5876-7-26 19366456 PMC2679712

[7] Dai P, Li Q, Huang D, Yuan Y, Kang D, Miller DT, et al SLC26A4 c.919-2A>G varies among Chinese ethnic groups as a cause of hearing loss. Genet Med. 2008;10(8):586–92. Epub 2008/07/22. 10.1097/GIM.0b013e31817d2ef1 . 18641518

[8] Russell JFSaDW, editor. Molecular Cloning:A Laboratry Manual.2002.

[9] Dale O, Salo M. The Helsinki Declaration, research guidelines and regulations: present and future editorial aspects. Acta Anaesthesiol Scand. 1996;40(7):771–2. Epub 1996/08/01. . 8874560 10.1111/j.1399-6576.1996.tb04530.x

[10] Stenson PD, Ball EV, Mort M, Phillips AD, Shiel JA, Thomas NS, et al Human Gene Mutation Database (HGMD): 2003 update. Hum Mutat. 2003;21(6):577–81. Epub 2003/05/20. 10.1002/humu.10212 . 12754702

[11] Yoshinaga-Itano C. Benefits of early intervention for children with hearing loss. Otolaryngol Clin North Am. 1999;32(6):1089–102. Epub 1999/10/16. S0030-6665(05)70196-1 [pii]. . 10523454 10.1016/s0030-6665(05)70196-1

[12] Liu Y, Ke X, Qi Y, Li W, Zhu P. Connexin26 gene (GJB2): prevalence of mutations in the Chinese population. J Hum Genet. 2002;47(12):688–90. Epub 2003/01/11. 10.1007/s100380200106 . 12522692

[13] Yan D, Park HJ, Ouyang XM, Pandya A, Doi K, Erdenetungalag R, et al Evidence of a founder effect for the 235delC mutation of GJB2 (connexin 26) in east Asians. Hum Genet. 2003;114(1):44–50. Epub 2003/09/25. 10.1007/s00439-003-1018-11450 035. 14505035

[14] Li H, Li Q, Chen Y. [A literature review of epidemiological studies on mutation hot spots of Chinese population with non-syndromic hearing loss]. Lin Chung Er Bi Yan Hou Tou Jing Wai Ke Za Zhi. 2012;26(13):589–94. Epub 2012/09/26. . 23002642

[15] Ross SA, Novak Z, Kumbla RA, Zhang K, Fowler KB, Boppana S. GJB2 and GJB6 mutations in children with congenital cytomegalovirus infection. Pediatr Res. 2007;61(6):687–91. Epub 2007/04/12. 10.1203/pdr.0b013e3180536609 . 17426645

[16] Yuan Y, Guo W, Tang J, Zhang G, Wang G, Han M, et al Molecular epidemiology and functional assessment of novel allelic variants of SLC26A4 in non-syndromic hearing loss patients with enlarged vestibular aqueduct in China. PLoS One. 2012;7(11):e49984 Epub 2012/11/28. 10.1371/journal.pone.0049984 PONE-D-12-23441 [pii]. 23185506 PMC3503781

[17] Liu Y, Wang L, Feng Y, He C, Liu D, Cai X, et al A New Genetic Diagnostic for Enlarged Vestibular Aqueduct Based on Next-Generation Sequencing. PLoS One. 2016;11(12):e0168508 Epub 2016/12/21. 10.1371/journal.pone.0168508 PONE-D-16-31811 [pii]. 27997596 PMC5173027

[18] Campbell C, Cucci RA, Prasad S, Green GE, Edeal JB, Galer CE, et al Pendred syndrome, DFNB4, and PDS/SLC26A4 identification of eight novel mutations and possible genotype-phenotype correlations. Hum Mutat. 2001;17(5):403–11. Epub 2001/04/24. 10.1002/humu.1116 [pii] . 11317356

[19] Saliba I, Gingras-Charland ME, St-Cyr K, Decarie JC. Coronal CT scan measurements and hearing evolution in enlarged vestibular aqueduct syndrome. Int J Pediatr Otorhinolaryngol. 2012;76(4):492–9. Epub 2012/01/28. S0165-5876(12)00034-1 [pii] 10.1016/j.ijporl.2012.01.004 . 22281371

[20] Wang QJ, Zhao YL, Rao SQ, Guo YF, Yuan H, Zong L, et al A distinct spectrum of SLC26A4 mutations in patients with enlarged vestibular aqueduct in China. Clin Genet. 2007;72(3):245–54. Epub 2007/08/28. CGE862 [pii] 10.1111/j.1399-0004.2007.00862.x . 17718863

[21] Yang T, Vidarsson H, Rodrigo-Blomqvist S, Rosengren SS, Enerback S, Smith RJ. Transcriptional control of SLC26A4 is involved in Pendred syndrome and nonsyndromic enlargement of vestibular aqueduct (DFNB4). Am J Hum Genet. 2007;80(6):1055–63. Epub 2007/05/16. S0002-9297(07)61024-6 [pii] 10.1086/518314 17503324 PMC1867094

[22] Bogazzi F, Russo D, Raggi F, Ultimieri F, Berrettini S, Forli F, et al Mutations in the SLC26A4 (pendrin) gene in patients with sensorineural deafness and enlarged vestibular aqueduct. J Endocrinol Invest. 2004;27(5):430–5. Epub 2004/07/29. 5341 [pii] 10.1007/BF03345286 . 15279074

[23] Lu J, Li Z, Zhu Y, Yang A, Li R, Zheng J, et al Mitochondrial 12S rRNA variants in 1642 Han Chinese pediatric subjects with aminoglycoside-induced and nonsyndromic hearing loss. Mitochondrion. 2010;10(4):380–90. Epub 2010/01/27. S1567-7249(10)00010-3 [pii] 10.1016/j.mito.2010.01.007 20100600 PMC2874659

[24] Zhao H, Li RH, Wang QJ, Yan QF, Deng JH, Han DY, et al Maternally inherited aminoglycoside-induced and nonsyndromic deafness is associated with the novel C1494T mutation in the mitochondrial 12S rRNA gene in a large chinese family. American Journal of Human Genetics. 2004;74(1):139–52. 10.1086/381133 ISI:000187723500013. 14681830 PMC1181901

[25] Smith RJ, Bale JF Jr., White KR. Sensorineural hearing loss in children. Lancet. 2005;365(9462):879–90. Epub 2005/03/09. S0140-6736(05)71047-3 [pii] 10.1016/S0140-6736(05)71047-3 . 15752533

[26] Wollnik B, Tukel T, Uyguner O, Ghanbari A, Kayserili H, Emiroglu M, et al Homozygous and heterozygous inheritance of PAX3 mutations causes different types of Waardenburg syndrome. Am J Med Genet A. 2003;122A(1):42–5. Epub 2003/09/02. 10.1002/ajmg.a.20260 . 12949970

[27] Tassabehji M, Newton VE, Liu XZ, Brady A, Donnai D, Krajewska-Walasek M, et al The mutational spectrum in Waardenburg syndrome. Hum Mol Genet. 1995;4(11):2131–7. Epub 1995/11/01. . 8589691 10.1093/hmg/4.11.2131

[28] Bondurand N, Dastot-Le Moal F, Stanchina L, Collot N, Baral V, Marlin S, et al Deletions at the SOX10 gene locus cause Waardenburg syndrome types 2 and 4. Am J Hum Genet. 2007;81(6):1169–85. Epub 2007/11/14. S0002-9297(07)63767-7 [pii] 10.1086/522090 17999358 PMC2276340

[29] Chen H, Jiang L, Xie Z, Mei L, He C, Hu Z, et al Novel mutations of PAX3, MITF, and SOX10 genes in Chinese patients with type I or type II Waardenburg syndrome. Biochem Biophys Res Commun. 2010;397(1):70–4. Epub 2010/05/19. S0006-291X(10)00957-5 [pii] 10.1016/j.bbrc.2010.05.066 . 20478267

[30] Xia JH, Liu CY, Tang BS, Pan Q, Huang L, Dai HP, et al Mutations in the gene encoding gap junction protein beta-3 associated with autosomal dominant hearing impairment. Nat Genet. 1998;20(4):370–3. Epub 1998/12/08. 10.1038/3845 . 9843210

[31] de Kok YJ, Bom SJ, Brunt TM, Kemperman MH, van Beusekom E, van der Velde-Visser SD, et al A Pro51Ser mutation in the COCH gene is associated with late onset autosomal dominant progressive sensorineural hearing loss with vestibular defects. Hum Mol Genet. 1999;8(2):361–6. Epub 1999/02/05. ddc041 [pii]. . 9931344 10.1093/hmg/8.2.361

[32] Yuan HJ, Han DY, Sun Q, Yan D, Sun HJ, Tao R, et al Novel mutations in the vWFA2 domain of COCH in two Chinese DFNA9 families. Clin Genet. 2008;73(4):391–4. Epub 2008/03/04. CGE972 [pii] 10.1111/j.1399-0004.2008.00972.x . 18312449

[33] Street VA, Kallman JC, Robertson NG, Kuo SF, Morton CC, Phillips JO. A novel DFNA9 mutation in the vWFA2 domain of COCH alters a conserved cysteine residue and intrachain disulfide bond formation resulting in progressive hearing loss and site-specific vestibular and central oculomotor dysfunction. Am J Med Genet A. 2005;139A(2):86–95. Epub 2005/11/02. 10.1002/ajmg.a.30980 . 16261627

[34] de Kok YJ, Cremers CW, Ropers HH, Cremers FP. The molecular basis of X-linked deafness type 3 (DFN3) in two sporadic cases: identification of a somatic mosaicism for a POU3F4 missense mutation. Hum Mutat. 1997;10(3):207–11. Epub 1997/01/01. 10.1002/(SICI)1098-1004(1997)10:3<207::AID-HUMU5>3.0.CO;2-F [pii] . 9298820

