# Supplementary material for: A rapid improved multiplex ligation detection reaction method for the identification of gene mutations in hereditary hearing loss
Source: PLoS One. 2019 Apr 11;14(4):e0215212. doi: 10.1371/journal.pone.0215212 (PMC6459514; doi:10.1371/journal.pone.0215212)
Supplement: S1 Table — (DOCX) [file pone.0215212.s001.docx]

**S1 Table. Primer sequences and concentration in PCR mixture 1**

| **Primer Name** | **Concentration (**µ**M)** | **Primer Sequence** |
| --- | --- | --- |
| SNP01F | 1 | AGAGACCCCAACGCCGAGAc |
| SNP01R | 1 | ggcccAAGGACGTGTGTTG |
| SNP02-08F | 1 | GCAAACCGCCCAGAGTAGAAGAT |
| SNP02-08R | 1 | TCCCCCTTGATGAACTTCCTCTT |
| SNP09-10F | 1 | TTGTCCCAACACTGTGGACTGC |
| SNP09-10R | 1 | ACTGGGCAATGCGTTAAACTGG |
| SNP11-13F | 1 | ACACTCTCTGGCATGGCTTCAA |
| SNP11-13R | 1 | ACCCTGTGGCAGATGAGGTAGC |
| SNP14F | 1 | TCTTGGAGTGGCTCCCCAAATA |
| SNP14R | 1 | GCAGCAGTTTCCCAGGAAGAGA |
| SNP15F | 1 | ACTGCTGGATTGCTCACCATTG |
| SNP15R | 1 | ACCCCCTTGGGATGGATTTAAC |
| SNP16-18F | 1 | AAATTGGACCACCACGCAGAGT |
| SNP16-18R | 1 | TCTGTTGCCATTCCTCGACTTG |
| SNP19F | 1 | GCTCCTCTGAGCAACTGTGACTTG |
| SNP19R | 1 | gaatgaaGTCTCAAAAGAGGTTAGAAAACA |
| SNP20F | 1 | GAACGTTCCCAAAGTGCCAATC |
| SNP20R | 1 | GCCCTGTTGCAATACTGGACAA |
| SNP21F | 1 | CGGGTTCTTTGACGACAACATT |
| SNP21R | 1 | AAATGGAACCTTGACCCTCTTGA |
| SNP22-23F | 1 | GGTCGAAGGTGGATTTAGCAGTAAAC |
| SNP22-23R | 1 | GGAGTGGGTTTGGGGCTAGGTT |
| SNP24F | 1 | CCACCACTATGCCCCAAGAAGT |
| SNP24R | 1 | GCCCCACATATGCTCGATACAG |
| SNP25F2 | 1 | GCTTTCTTCACAAGAGAGTTCACAGG |
| SNP25R2 | 1 | AAAATGTTACCATTATTGCTGGGATTC |
| SNP26F | 1 | GGAGCCCGACCAGCATTG |
| SNP26R | 1 | TCTGTTTTCACGGTGTGCGAATA |
| SNP27F | 1 | CCTGACAATTCGCCCAAACAA |
| SNP27R | 1 | CCGGGAATGAGATGGTTGAAAG |
| SNP28\|-/aagF | 1 | CCGTTGTCATGACCTGGAGAAGT |
| SNP28\|-/aagR | 1 | ACGCGATTGTACTCACGGATCA |
| SNP29\|-/GF | 1 | CGTTGGACTCTTTGCGAGGAC |
| SNP29\|-/GR | 1 | TCCTGCTGCTCCTTCTTGACCT |
| SNP30-31F | 1 | CTCTTGCCCCCACAGAGATCC |
| SNP30-31R | 1 | GGGTTCCACACCTCACACTGA |
