# Supplementary material for: A rapid improved multiplex ligation detection reaction method for the identification of gene mutations in hereditary hearing loss
Source: PLoS One. 2019 Apr 11;14(4):e0215212. doi: 10.1371/journal.pone.0215212 (PMC6459514; doi:10.1371/journal.pone.0215212)
Supplement: S2 Table — (DOCX) [file pone.0215212.s002.docx]

**S2 Table. Oligo sequences and concentration in labeling oligo mixture**

| **Name** | **Concentration (μM)** | **Sequence** |
| --- | --- | --- |
| FamLabel | 3 | [FAM]AGATTCGCAATGGCCAGCACA |
| VICLabel | 3 | [VIC]GCTTGGGCAATACTCGCTTAT |
| NEDLabel | 3 | [NED]TATCCGACTGCGCACGTTATT |
| PETLabel | 3 | [PET]GTTGGGCGCTAGATCAATCGT |
| FAMTem | 1 | ATATCAGTCCGAACGCGGAATGTGCTGGCCATTGCGAATCT |
| VICTem | 1 | CAGGAGCCCGAATAACCGTAATAAGCGAGTATTGCCCAAGC |
| NEDTem | 1 | GGACGAATTGACCCGAGAGAAATAACGTGCGCAGTCGGATA |
| PETTem | 1 | ACTAATCCGGCCCACGAACAACGATTGATCTAGCGCCCAAC |
|  |  |  |
