# Supplementary material for: A rapid improved multiplex ligation detection reaction method for the identification of gene mutations in hereditary hearing loss
Source: PLoS One. 2019 Apr 11;14(4):e0215212. doi: 10.1371/journal.pone.0215212 (PMC6459514; doi:10.1371/journal.pone.0215212)
Supplement: S3 Table — (DOCX) [file pone.0215212.s003.docx]

**S3 Table. Probe sequences and concentration in probe mixture**

| **Probe Name** | **Target Allele** | **Concentration (μM)** | **Probe Sequence (5' phosphorated)** |
| --- | --- | --- | --- |
| SNP01RG | G | 1 | TTCCGCGTTCGGACTGATATTCCGGGGCCGGCGGGCTCAC |
| SNP01RA | A | 1 | TACGGTTATTCGGGCTCCTGTTCCGGGGCCGGCGGGCTGAT |
| SNP01RP |  | 2 | CTGCGTCGGGAGGAAGCGCGTTTTTTT |
| SNP02FD | - | 1 | TACGGTTATTCGGGCTCCTGTGCTGCAGACGATCCTGGGGG |
| SNP02FI | G | 1 | TTCCGCGTTCGGACTGATATCTGCAGACGATCCTGGGGGG |
| SNP02FP |  | 2 | TGTGAACAAACACTCCACCAGCTTTTTTTTTTTTTTTTTTTT |
| SNP03FA | A | 1 | TACGGTTATTCGGGCTCCTGTCTCCACCAGCATTGGAAAGATGTA |
| SNP03FG | G | 1 | TTCCGCGTTCGGACTGATATCTCCACCAGCATTGGAAAGATGTG |
| SNP03FP |  | 2 | GCTCACCRTCCTCTTCATTTTTCTTTTTTTTTTTTTTTTTT |
| SNP04FG | G | 1 | TCTCTCGGGTCAATTCGTCCTTGCAAAGGAGGTGTGGGGAGTTG |
| SNP04FP |  | 1 | AGCAGGCCGACTTTGTCTGCTTTTTTTTTTTTTTTTTTTTTTTTTT |
| SNP04FT | T | 2 | TGTTCGTGGGCCGGATTAGTGCAAAGGAGGTGTGGGGAGCTT |
| SNP05FD | - | 1 | TACGGTTATTCGGGCTCCTGTCCGACTTTGTCTGCAACACCC |
| SNP05FI | T | 1 | TTCCGCGTTCGGACTGATATCGACTTTGTCTGCAACACCCT |
| SNP05FP |  | 2 | GCAGCCAGGCTGCAAGAACGTTTTTTTTTTTTTTTTTTTTTTTTTTTTTTTTTTTT |
| SNP06FD | - | 1 | TACGGTTATTCGGGCTCCTGTCTGCAACACCCTGCAGCCAG |
| SNP06FI | gctgcaagaacgtgtg | 1 | TTCCGCGTTCGGACTGATATCCAGGCTGCAAGAACGTGTG |
| SNP06FP |  | 2 | CTACGATCACTACTTCCCCATCTCCTTTTTTTTTTTTTTTTTTTTTTTTTTTTTTTTTTTTTT |
| SNP07FD | - | 1 | TGTTCGTGGGCCGGATTAGTCCCACATCCGGCTATGGGCC |
| SNP07FI | C | 1 | TCTCTCGGGTCAATTCGTCCTTCCACATCCGGCTATGGGCCC |
| SNP07FP |  | 2 | TGCAGCTGATCTTCGTGTCCATTTTTTTTTTTTTTTTTTTTTTTTTTTTTTTTTTTTTTTTTT |
| SNP08FD | - | 1 | TACGGTTATTCGGGCTCCTGTTTTTTTGCACGTGGCCTACCGGAGAC |
| SNP08FI | AT | 1 | TTCCGCGTTCGGACTGATATTTTTTTACGTGGCCTACCGGAGACAT |
| SNP08FP |  | 2 | GAGAAGAAGAGGAAGTTCATCAAGGGTTTTTTTTTTTTTTTTTTTTTTTTTTTTTTTTTT |
| SNP09FC | C | 1 | TCTCTCGGGTCAATTCGTCCTTACACTGCAATCATGAACACTGTCAG |
| SNP09FT | T | 1 | TGTTCGTGGGCCGGATTAGTACACTGCAATCATGAACACTGTCAA |
| SNP09FP |  | 2 | GACAGTCTTCTCCGTGGGCCGTTTTTTTTTTTTTTTTTTTTTTTTTTTTTTT |
| SNP10FC | C | 1 | TCTCTCGGGTCAATTCGTCCTTATTCAGCAGGRTGCAAATTCAAG |
| SNP10FT | T | 1 | TGTTCGTGGGCCGGATTAGTATTCAGCAGGRTGCAAATTCGAA |
| SNP10FP |  | 2 | ACACTGCAATCATGAACACTGTGAATTTTTTTTTTTTTTTTTTTTTTTTTT |
| SNP11FC | C | 1 | TTCCGCGTTCGGACTGATATTCGTGGACTGCTACATTGACC |
| SNP11FT | T | 1 | TACGGTTATTCGGGCTCCTGTTCGTGGACTGCTACATTGACT |
| SNP11FP |  | 2 | GACCTACCGAGAAGAAAATCTTCACCTACTTCATTTTTTTTTTTTTTTTTTTTTTTTTT |
| SNP12FA | A | 1 | TGTTCGTGGGCCGGATTAGTACTGCTACATTGCCCGACCTAGCA |
| SNP12FG | G | 1 | TCTCTCGGGTCAATTCGTCCTTACTGCTACATTGCCCGACCTAACG |
| SNP12FP |  | 2 | AGAAGAAAATCTTCACCTACTTCATGGTGTTTTTTTTTTTTTTTTTTTTTTTTTTT |
| SNP13FA | A | 1 | TACGGTTATTCGGGCTCCTGTTCACCTACTTCATGGTGGTCA |
| SNP13FG | G | 1 | TTCCGCGTTCGGACTGATATTCACCTACTTCATGGTGGTCG |
| SNP13FP |  | 2 | CCTCCGCCGTCTGCATCGTATTTTTTTTT |
| SNP14RC | C | 1 | TCTCTCGGGTCAATTCGTCCTTCAGCGTGGCCACTAGCCAAG |
| SNP14RT | T | 1 | TGTTCGTGGGCCGGATTAGTCAGCGTGGCCACTAGCCGAA |
| SNP14RP |  | 2 | TACTAACTCCCGAAATGACGTCACTAATTTTTT |
| SNP15RA | A | 1 | TGTTCGTGGGCCGGATTAGTTGAAATGGCAGTAGCAATTATCGACT |
| SNP15RG | G | 1 | TCTCTCGGGTCAATTCGTCCTTTGAAATGGCAGTAGCAATTATCGACC |
| SNP15RP |  | 2 | GAAATAAAACAAAAGATGTTAAAAACTCCATTGTTTTTT |
| SNP16FA | A | 1 | TCTCTCGGGTCAATTCGTCCTTTCATTGCCTTTGGGATCACCA |
| SNP16FT | T | 1 | TGTTCGTGGGCCGGATTAGTTCATTGCCTTTGGGATCACCT |
| SNP16FP |  | 2 | ACATCTTCTCAGGATTCTTCTCTTGTTTTTTTTTT |
| SNP17FA | A | 1 | TACGGTTATTCGGGCTCCTGTGCCACCACTGCTCTTTCACA |
| SNP17FG | G | 1 | TTCCGCGTTCGGACTGATATGCCACCACTGCTCTTTCACG |
| SNP17FP |  | 2 | CACGGCCGTCCAGGAGAGCATTTTTTTTTTTTTTTTTTTTTTTTTTTTTTT |
| SNP18FC | C | 1 | TCTCTCGGGTCAATTCGTCCTTCCACCACTGCTCTTTCCCGAAC |
| SNP18FT | T | 1 | TGTTCGTGGGCCGGATTAGTCCACCACTGCTCTTTCCCGGAT |
| SNP18FP |  | 2 | GGCCGTCCAGGAGAGCACTGTTTTTTTTTTTTTTTTTTTTTTTTTTTTT |
| SNP19RA | A | 1 | TACGGTTATTCGGGCTCCTGTAGAGGTTAGAAAACAAATTTCTAGGGATAAAAGAT |
| SNP19RG | G | 1 | TTCCGCGTTCGGACTGATATAGAGGTTAGAAAACAAATTTCTAGGGATAAAAGAC |
| SNP19RP |  | 2 | TTACTGTGGACTTGATACATTTTTTAAAACCAT |
| SNP20RA | A | 1 | TCTCTCGGGTCAATTCGTCCTTCCAGAACCTTACCACCCTCT |
| SNP20RT | T | 1 | TGTTCGTGGGCCGGATTAGTCCAGAACCTTACCACCCACA |
| SNP20RP |  | 2 | GTGATCTCACTCCAACAACGTCCTTTTTTT |
| SNP21FA | A | 1 | TGTTCGTGGGCCGGATTAGTGACACATTCTTTTTGACGGTGCA |
